# *De novo *assembly and characterization of root transcriptome using Illumina paired-end sequencing and development of cSSR markers in sweetpotato (*Ipomoea batatas*)

**DOI:** 10.1186/1471-2164-11-726

**Published:** 2010-12-24

**Authors:** Zhangying Wang, Boping Fang, Jingyi Chen, Xiongjian Zhang, Zhongxia Luo, Lifei Huang, Xinliang Chen, Yujun Li

**Affiliations:** 1 Crops Research Institute, Guangdong Academy of Agricultural Sciences, Guangzhou, 510640 PR China

## Abstract

**Background:**

The tuberous root of sweetpotato is an important agricultural and biological organ. There are not sufficient transcriptomic and genomic data in public databases for understanding of the molecular mechanism underlying the tuberous root formation and development. Thus, high throughput transcriptome sequencing is needed to generate enormous transcript sequences from sweetpotato root for gene discovery and molecular marker development.

**Results:**

In this study, more than 59 million sequencing reads were generated using Illumina paired-end sequencing technology. *De novo *assembly yielded 56,516 unigenes with an average length of 581 bp. Based on sequence similarity search with known proteins, a total of 35,051 (62.02%) genes were identified. Out of these annotated unigenes, 5,046 and 11,983 unigenes were assigned to gene ontology and clusters of orthologous group, respectively. Searching against the Kyoto Encyclopedia of Genes and Genomes Pathway database (KEGG) indicated that 17,598 (31.14%) unigenes were mapped to 124 KEGG pathways, and 11,056 were assigned to metabolic pathways, which were well represented by carbohydrate metabolism and biosynthesis of secondary metabolite. In addition, 4,114 cDNA SSRs (cSSRs) were identified as potential molecular markers in our unigenes. One hundred pairs of PCR primers were designed and used for validation of the amplification and assessment of the polymorphism in genomic DNA pools. The result revealed that 92 primer pairs were successfully amplified in initial screening tests.

**Conclusion:**

This study generated a substantial fraction of sweetpotato transcript sequences, which can be used to discover novel genes associated with tuberous root formation and development and will also make it possible to construct high density microarrays for further characterization of gene expression profiles during these processes. Thousands of cSSR markers identified in the present study can enrich molecular markers and will facilitate marker-assisted selection in sweetpotato breeding. Overall, these sequences and markers will provide valuable resources for the sweetpotato community. Additionally, these results also suggested that transcriptome analysis based on Illumina paired-end sequencing is a powerful tool for gene discovery and molecular marker development for non-model species, especially those with large and complex genome.

## Background

Sweetpotato (*Ipomoea batatas*) is a hexaploid (2n = 6x = 90) dicot and belongs to the family of *Convolvulaceae*. It is one of the world's important food crops, especially in developing countries. The tuberous roots of sweetpotato are usually used as staple food, animal feed, industrial material or raw material for alcohol production. According to the Food and Agriculture Organization (FAO) statistics, the world production of sweetpotato in 2008 was more than 110 million tons, and the majority came from China, with a production of around 85 million tons from about 3.7 million hectares [1]. Due to the high sink potential of the tuberous root, sweetpotato has one of the highest dry matter productivity rates among crops [2,3]. In addition to its agricultural importance, the sweetpotato tuberous root, involved in carbohydrate storage and vegetative propagation, is also a unique organ, which has the value of biological research for organogenesis and evolution. Therefore, understanding the processes regulating the root formation and development is of particular importance. During the last decade, a large number of transcriptomic and genomic sequences became available in model organisms, such as *Arabidopsis*, *Antirrhinum *and rice, which have greatly improved the understanding of the complexity of growth and development in higher plants. For sweetpotato, a total of 22, 731 EST sequences have been deposited in GenBank database (as of June 2010). After trimming and assembly, only 3,407 contigs and 4,856 singletons were obtained (unpublished data). However, the tuberous root formation and development of sweetpotato are complex biological processes involving morphogenesis as well as dry matter accumulation. The publicly available data are not sufficient for elucidating the molecular mechanisms controlling the traits of interest, and moreover, with traditional methods sequencing of these randomly selected cDNA clones from various tissues often has insufficient coverage of less-abundant transcripts, which usually play irreplaceable functions. In addition, to date, only about 300 SSR markers were developed for sweetpotato [4-6]. EST collections will also facilitate the development of molecular markers for further genetic research in this and related species. Therefore, extensive genomic and transcriptomic sequence data are needed for sweetpotato, which can be used to discover new genes related to tuberous root formation and development, and can also make it possible to construct high density microarrays for further characterization of gene expression profiles during these processes.

However, given that cultivated sweetpotato is a hexaploid outbreeding species with a large genome (2, 205 Mb) [7] and a high degree of heterozygosity, the prohibitive costs associated with sequencing and assembling such a large and complex genome make it infeasible to consider whole genome sequencing in the near future. Fortunately, transcriptome sequencing is an attractive alternative to the whole genome sequencing. It is well known that the majority of most eukaryotic genomes are composed of non-coding DNA, and transcribed sequences excluding introns contain a high content of functional information [8]. Furthermore, large collections of ESTs have proven invaluable for functional genomics and molecular marker development [9-13]. Currently, however, traditional sequencing methods for the generation of ESTs require costly and time-consuming approaches involving cDNA library construction, cloning, and labor intensive Sanger sequencing. The newly developed high throughput sequencing technology, i.e. Next Generation Sequencing (NGS), including the Roche/454 Genome Sequencer FLX Instrument, the ABI SOLiD System, and the Illumina Genome Analyser, is a powerful and cost-efficient tool for advanced research in many areas, including re-sequencing, microRNA expression profiling, DNA methylation, especially *de novo *transcriptome sequencing for non-model organisms [10,14-24]. Over the past several years, NGS has greatly accelerated our understanding of the complexity of gene expression, regulation and networks in model and non-model organisms. Though the transcriptome sequencing for non-model organisms using NGS was almost confined to 454 pyrosequencing due to its longer read length compared with the other two platforms [10,20,25], it is noteworthy that a draft genome sequence for the giant panda has been generated and assembled successfully using only Illumina Genome Analyser sequencing technology [26]. Recently whitefly transcriptome was also characterized using this short read sequencing platform [27].

In the present study, we utilized Illumina paired-end sequencing technology to characterize the root transcriptome of sweetpotato and to develop EST-derived SSR markers. Non-normalized cDNA collections from different types of roots were used to generate a broad survey of genes associated with tuberous root formation and development. To the best of our knowledge, this study is the first exploration to characterize the root transcriptome of sweetpotato through the analysis of large-scale transcript sequences resulting from Illumina paired-end sequencing. In addition to offering valuable sequence resource to sweet potato community, our objective was also to provide an efficient, inexpensive and reliable approach for transcriptome sequencing that can be readily adopted by researchers studying non-model organisms.

## Results

### Illumina paired-end sequencing and *de novo *assembly

With the purpose of generating a broad survey of genes associated with tuberous root formation and development, RNA was extracted from fibrous roots, pencil roots and tuberous roots at three developmental stages. Using Illumina paired-end sequencing technology, each sequencing feature can yield 2 × 75 bp independent reads from either end of a DNA fragment. In this study, a total of 59,233,468 raw sequencing reads with the length of 75 bp were generated from a 200 bp insert library. An assembler, SOAPdenovo http://soap.genomics.org.cn developed specifically for use with next-generation short-read sequences, was employed for *de novo *assembly. After stringent quality check and data cleaning, approximately 51 million high-quality reads were obtained with 99.30% Q20 bases (base quality more than 20). Based on the high quality reads, a total of 208,127 contigs were assembled with an average length of 202 bp. The length of contigs ranged from 75 to 6,891 bp. Contigs with length more than 100 bp accounted for 72.4% (Table 1).

**Table 1:** Length distribution of assembled contigs, scaffolds and unigenes

Nucleotides length (bp)	contigs	Scaffolds	unigenes
75-100	57424	1303	0
101-200	95153	46037	280
201-300	23619	20788	20632
301-400	11590	9987	9949
401-500	6527	6311	6302
501-600	3979	4040	4028
601-700	2737	2844	2844
701-800	1866	2010	2014
801-900	1307	1612	1621
901-1000	901	1236	1241
1001-1200	1166	1864	1862
1201-1400	714	1325	1322
1401-1600	434	1027	1029
1601-1800	268	824	824
1801-2000	174	597	593
2001-2200	98	455	458
2201-2400	61	334	333
2401-2600	35	258	261
2601-2800	21	197	199
2801-3000	18	159	157
>3000	35	567	567

Total	208,127	103,775	56,516
Minimum length (bp)	75	100	200
Maximum length (bp)	6891	10679	10679
N50 (bp)	252	585	765
Average length (bp)	202	377	581
Total Nucleotides length (bp)	42,074,974	39,129,156	32,852,951

**Figure 1:**
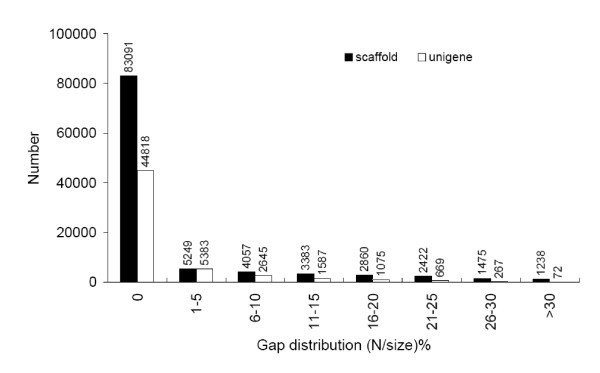
**Gap distribution of assembled scaffolds and unigenes**. Gap distribution (N/size) %: gap percentage (N amount/sequence length) distribution.

With paired-end reads, it is possible to identify contigs derived from the same transcript as well as the distances between these contigs. We, therefore, mapped the reads back to contigs, and then with paired-end information joined contigs into scaffolds using "N" to represent unknown nucleotides between each two contigs. As a result, 103,775 scaffolds were obtained with an average length of 377 bp (table 1). Scaffolds with the length ranging from 100 to 400 bp accounted for 75.28%. Although 80.07% scaffolds had not a gap at all (Figure 1), roughly 1.28 Mb gaps (3.27% of total unigene sequences) remained unclosed.

To further shorten the remaining gaps, we gathered the paired-end reads with one end mapped on the unique contig and the other end located in the gap region and performed local assembly with the unmapped end to fill in the small gaps within the scaffolds. Such sequences containing least Ns and not being extended on either end were defined as unigenes. In this step, more than half of gaps were filled, and only 0.52 Mb gaps (1.60% of total unigene sequences) remained unclosed. The gap distribution for unigenes was shown in Figure 1. Finally the *de novo *assembly yielded 56,516 unigenes with an average length of 581 bp and a total length of 32.85 Mb (Table 1). The length of assembled unigenes ranged from 200 to 10,679 bp. There were 30,861 unigenes (54.61%) with length varying from 200 to 400 bp, 18,050 unigenes (31.94%) in the length range of 401 to 1000 bp, and 7,605 unigenes (13.46%) with length more than 1000 bp (Table 1).

**Figure 2:**
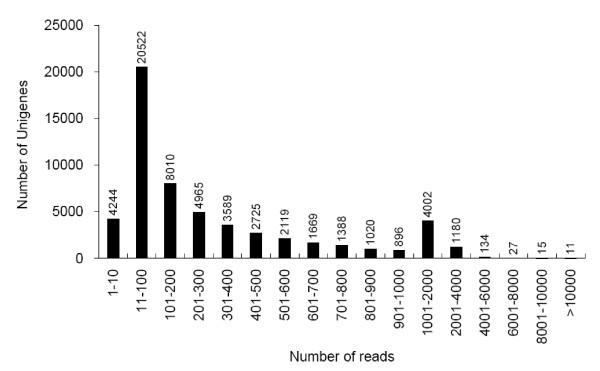
**Assessment of assembly quality**. Distribution of unique-mapped reads of the assembled unigenes.

**Figure 3:**
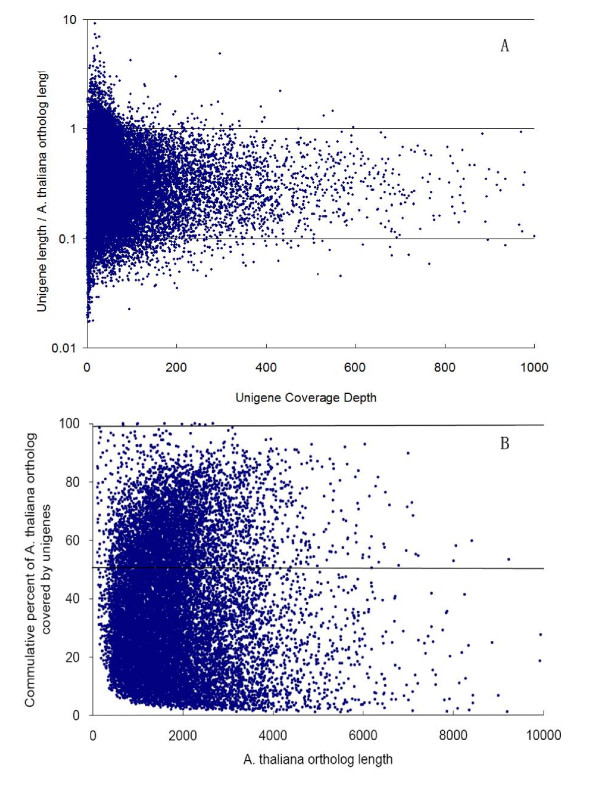
**Comparison of *I. batatas *unigenes to orthologous *A. thaliana *coding sequences**. (A) The ratio of *I. batatas *unigene length to *A. thaliana *ortholog length was plotted against *I. batatas *unigene coverage depth. (B) Total percent of *A. thaliana *ortholog coding sequence that was covered by all *I. batatas *unigenes. In total, 502 orthologs could be covered by unigenes with a percentage more than 80%, and the cover percentage of around 5,000 orthologs ranged from 50-80%. Additionally, 27% orthologs were covered with only 20% or lower.

To evaluate the quality and coverage of the assembled unigenes, all the usable sequencing reads were realigned to the unigenes using SOAPaligner [28], allowing up to 2 base mismatches. The sequencing depth ranged from 0.1 to 4,079 folds, with an average of 48.36 folds. About 92.5% of the unigenes were realigned by more than 10 reads, 56.2% were remapped by more than 100 reads, and almost 10% were realigned by more than 1000 reads (Figure 2). To further assess the extent of transcript coverage provided by unigenes and to evaluate how coverage depth affected the assembly of unigenes, we plotted the ratio of assembled unigene length to *A. thaliana *ortholog length against coverage depth (Figure 3A). Most of *A. thaliana *ortholog coding region can be covered by our individual unigenes, although a large number of deeply covered unigenes failed to cover the complete coding regions of their *A. thaliana *orthologs. It is worth noting that, to a certain extent, increased coverage depth can result in higher coverage of the coding regions. Actually, in many cases, multiple unigenes covered different regions of *A. thaliana *orthologs. Plotting the summed proportion of *A. thaliana *orthologs covered by all Illumina unigenes showed that 502 orthologs could be covered by unigenes with a percentage more than 80%, and the cover percentage of around 5,000 orthologs ranged from 50-80%. Additionally, 27% orthologs were covered with only 20% or lower (Figure 3B). The results indicated that additional sequencing would be needed for more comprehensive transcriptome coverage.

### Functional annotation by searching against public databases

**Figure 4:**
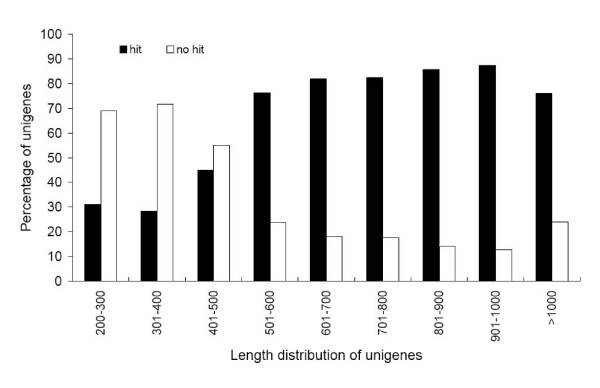
**Comparison of unigene length between hit and no-hit unigenes**. Longer contigs were more likely to have BLAST matches in protein databases. In this study, 79% of unigenes over 500 bp in length had BLAST matches, whereas only 30% of unigenes shorter than 300 bp did.

**Figure 5:**
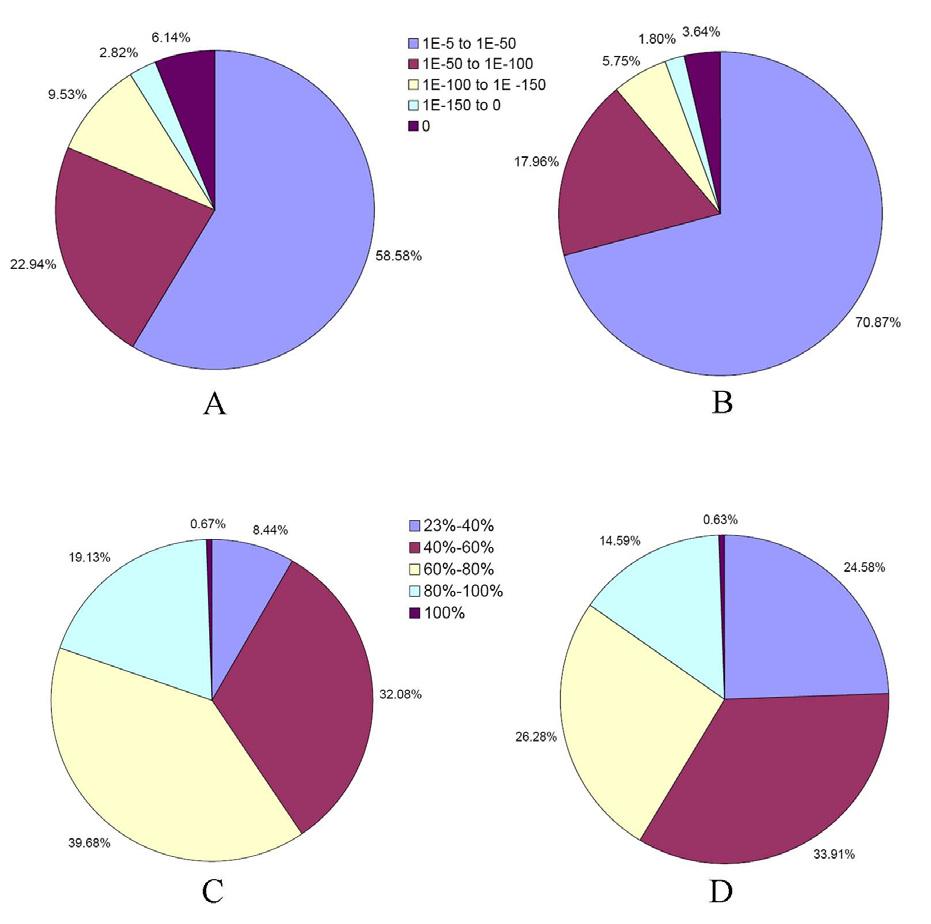
**Characteristics of similarity search of unigenes against Nr and Swiss-Prot databases**. (A) *E-value *distribution of BLAST hits for each unigene with a cutoff *E-value *of 1.0E-5 in Nr database. (B) *E-value *distribution of BLAST hits for each unigene with a cutoff *E-value *of 1.0E-5 in Swiss-Prot database. (C) Similarity distribution of the top BLAST hits for each unigene in Nr database. (D) Similarity distribution of the top BLAST hits for each unigenes in Swiss-Prot dababase.

For validation and annotation of assembled unigenes, sequence similarity search was conducted against the NCBI non-redundant protein (Nr) database and the Swiss-Prot protein database using BLASTx algorithm [29,30] with an *E *value threshold of 10^-5^. The results indicated that out of 56,516 unigenes, 27,435 (48.54%) showed significant similarity to known proteins in Nr database and matched 18,496 unique protein accessions. As expected, the similar percentage was found for the search against Swiss-Prot database. Of all the unigenes, 26,287 (46.21%) had BLAST hits in Swiss-Prot database and matched 11,914 unique protein accessions. Compared with [27], in which only 16.2% had BLAST hits in Nr database, the higher percentage in this study was partially due to the higher frequency of long sequences in our unigenes (581 bp average length versus 266 bp average length of whitefly) [27]. As reported by [10], the longer contigs were more likely to have BLAST matches in the protein databases. Our results also showed that 79% of unigenes over 500 bp in length had BLAST matches, whereas only 30% of unigenes shorter than 300 bp did (Figure 4). The *E-value *distribution of the top hits in the Nr database revealed that 41.42% of the mapped sequences showed significant homology (less than 1.0E-50), and nearly 20% of the sequences with greater than 80% similarity were found (Figure 5A and 5C). The *E-value *and similarity distributions of the top hits in the Swiss-Prot database had a comparable pattern with 30% and 15% of the sequences possessing significant homology and similarity, respectively (Figure 5B and 5D). Altogether, BLAST searches identified a total of 20,755 unique protein accessions, indicating that in this study the Illumina paried-end sequencing project generated a substantial fraction of sweetpotato genes.

Of all the 20,755 unigenes, 26 were uniquely mapped by more than 8,000 reads, which represented the most abundant transcripts in sweetpotato root cDNA library (Additional file 1, Table S1). Since sweetpotato tuberous root contains approximately 70% starch of the total dry weight, it is not surprising that some transcripts encoding the enzymes involved in starch metabolism were highly expressed, such as plant glycogenin-like starch initiation protein 1, ADP-glucose pyrophosphorylase *beta *subunit, granule bound starch synthase I, *alpha*-1, 4 glucan phosphorylase L isozyme and *Beta*-amylase [31-34]. Besides the high starch content, sweetpotato tuberous root also contains plenty of other components, such as alkaloid and vitamin C (Ascorbic Acid). Therefore, we also found two transcripts were highly expressed, one encoding the putrescine methyltransferase, which participates in alkaloid biosynthesis [35]; the other encoding the GDP-D-mannose 3,5-epimerase (GME), which is generally considered to be a key enzyme of the major ascorbate biosynthesis pathway in higher plants through converting GDP-d-mannose to GDP-l-galactose [36]. Notably, we found an abundant transcript encoding Rac-like GTP-binding protein, which was preferentially expressed at the tip of root hairs and believed to be involved in cell polarity control during the actin-dependent tip growth of root hairs [37,38]. Some transcripts encoding superoxide dismutase and metallothionein-like protein were also highly expressed, and these enzymes may play a role in the defense system or keeping metal homeostasis or detoxification [39-41]. In addition, abundant transcripts encoding ribosomal proteins and Like-Sm ribonucleoprotein (LSM)-related were also identified. However, it is noteworthy that the two most abundant transcripts, which had 49,469 and 30,626 reads mapped, respectively, showed no significant similarity to any known gene.

### Functional classification by GO and COG

**Figure 6:**
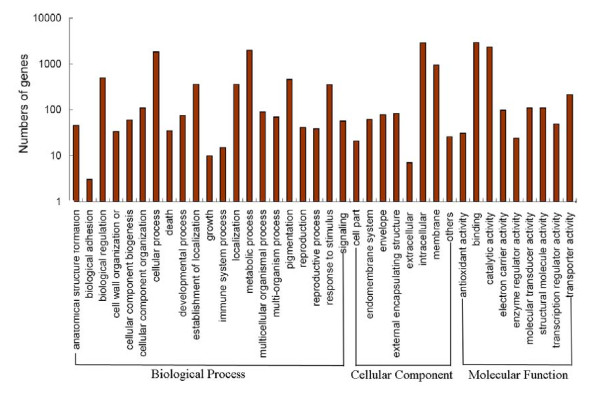
**Gene Ontology classification of assembled unigenes**. The results are summarized in three main categories: Biological process, Cellular component and Molecular function. In total, 5,046 unigenes with BLAST matches to known proteins were assigned to gene ontology.

Gene Ontology (GO) is an international standardized gene functional classification system which offers a dynamic-updated controlled vocabulary and a strictly defined concept to comprehensively describe the properties of genes and their products in any organism. GO has three ontologies: Molecular function, Cellular component and Biological process. On the basis of Nr annotation, the Blast2GO program [42] was used to obtain GO annotation for unigenes annotated by Nr. Then the WEGO software [43] was used to perform GO functional classification for these unigenes. In total, 5,046 unigenes with BLAST matches to known proteins were assigned to gene ontology classes with 16,595 functional terms. Of them, assignments to the biological process made up the majority (6,547, 39.45%) followed by molecular function (5,882, 35.44%) and cellular component (4,166, 25.10%, Figure 6).

The assigned functions of unigenes covered a broad range of GO categories. Under the biological process category, metabolic process (1,994 unigenes, 30.46%) and cellular process (1,837 unigenes, 28.06%) were prominently represented, indicating that some important metabolic activities occur in sweetpotato root. Interestingly, 459 unigenes were assigned to the pigmentation category. It was also noteworthy that a large number of genes (352 unigenes) involved in response to different stimulus. Under the category of molecular function, binding (2,904 unigenes, 49.37%) and catalytic (2,340 unigenes, 39.78%) represented the majorities of the category. Among the 2,904 unigenes assigned to the binding part, protein binding (679 unigenes) represented the most abundant classification, followed by ion binding (563 unigenes), ATP binding (521 unigenes), DNA binding (350 unigenes) and RNA binding (284 unigenes) (data not shown). For the cellular component category, 2,850 unigenes were located into intracellular, whereas only a few genes were assigned to extracellular region, macromolecular complex and virion.

**Figure 7:**
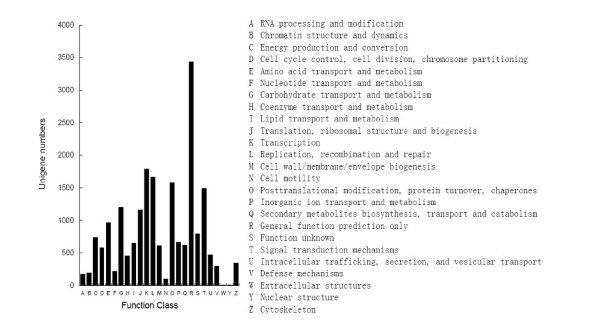
**Histogram presentation of clusters of orthologous groups (COG) classification**. All unigenes were aligned to COG database to predict and classify possible functions. Out of 27,435 Nr hits, 11,983 sequences were assigned to 25 COG classifications.

The Cluster of Orthologous Groups (COG) database is a database where the orthologous gene products were classified. Every protein in COG is assumed to be evolved from an ancestor protein, and the whole database is built on coding proteins with complete genome as well as system evolution relationships of bacteria, algae and eukaryotes. All unigenes were aligned to the COG database to predict and classify possible functions. Out of 27,435 Nr hits, 11,983 sequences were assigned to the COG classifications (Figure 7). Among the 25 COG categories, the cluster for General function prediction only (3,432, 17.01%) represented the largest group, followed by Transcription (1,789, 8.87%), Replication, recombination and repair (1,665, 8.25%), Posttranslational modification, protein turnover and chaperones (1,577, 7.82%), Signal transduction mechanisms (1,487, 7.37%), Carbohydrate transport and metabolism (1,200, 5.95%) and Translation, ribosomal structure and biogenesis (1,161, 5.75%), whereas only a few unigenes were assigned to Nulcear structure and Extracellular structure. In addition, 619 unigenes were assigned to Secondary metabolites biosynthesis, transport and catabolism (Figure 7).

### Functional classification by KEGG

**Figure 8:**
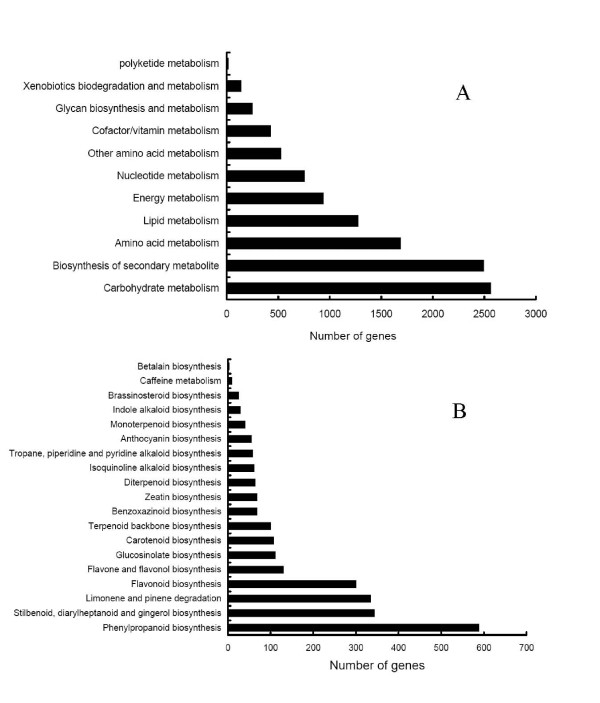
**Pathway assignment based on KEGG**. **(A) **Classification based on metabolism categories; **(B)** Classification based on secondary metabolite categories.

The Kyoto Encyclopedia of Genes and Genomes (KEGG) Pathway database records the networks of molecular interactions in the cells, and variants of them specific to particular organisms. Pathway-based analysis helps to further understand the biological functions and interactions of genes. Firstly, Based on a comparison against the KEGG database using BLASTx with an *E-value *cutoff of <10^-5^, out of the 56,516 unigenes, 17,598 (31.14%) had significant matches in the database and were assigned to 124 KEGG pathways. Among them, 11,056 unigenes having enzyme commission (EC) numbers were assigned to the metabolic pathways. As shown in Figure 8A, the KEGG metabolic pathways contained carbohydrate metabolism, the biosynthesis of secondary metabolite, amino acid metabolism, lipid metabolism and energy metabolism. In the secondary metabolism, 2,493 unigenes were classified into 19 subcategories, and most of them were mapped to phenylpropanoid biosynthesis, stilbenoid, diarylheptanoid and gingerol biosynthesis, limonene and pinene degradation, and flavonoid biosynthesis (Figure 8B). Surprisingly, in the KEGG map, most enzymes were mapped to the unigenes. These results indicated the active metabolic processes in sweetpotato root, but also implies that a variety of metabolites are synthesized in the root, suggesting that it, besides storing starch, is a valuable food source with various nutrients, such as complex carbohydrates, dietary fiber, *beta *carotene, vitamins, flavonoid and isoflavonoid, anthocyanin and alkaloid.

In addition to the genes assigned to the metabolism pathways, 3,205 unigenes were sorted to the genetic information processing involving transcription, translation, folding, sorting, degradation, replication and repair, and about 700 unigenes were classified into membrane transport, signal transduction, immune system and environmental adaptation. The results demonstrated the powerful ability of high-throughput sequencing to identify novel genes in non-model organisms, and these annotations also provided a valuable resource for investigating specific processes, functions and pathways involved in root formation and development.

### Development and characterization of cDNA-derived SSR markers

For further assessment of the assembly quality and development of new molecular markers, all of the 56,516 unigenes generated in this study were used to mine potential microsatellites which were defined as di- to hexanucleotide SSR with a minimum of four repetitions for all motifs. Using the MISA Perl script http://pgrc.ipk-gatersleben.de/misa/, a total of 4,114 potential cSSRs were identified in 3,594 unigenes, of which, 423 sequences contained more than 1 cSSR, and 275 cSSRs were present in compound form (Table 2). In order to identify the putative function of genes containing the cSSR loci, 3,594 unigenes were searched against UniProt database http://www.uniprot.org with *E*-value cutoff less than 10^-5^. Among them, 2,266 unigenes had BLAST hits to known proteins in this database. Based on the cSSR-containing sequences, 100 pairs of cSSR primers were designed using Primer Premier 6.0 (PREMIER Biosoft International, Palo Alto CA). The detailed information of designed primers is shown in Additional file 2, Table S2. Of the 100 designed cSSRs, 44 were found in the coding regions, 21 in the 5' untranslated regions (5' UTR), 13 in the 3' UTR and 22 in those genes without any hit to known proteins. Among the 100 primer pairs, 92 primer pairs were successful in PCR amplification in cultivated sweetpotato. The remaining 8 primers failed to generate PCR products at various annealing temperatures and Mg^2+ ^concentrations and would be excluded from further analysis. Of the 92 working primer pairs, 47 amplified PCR products at the expected sizes, and 12 primer pairs resulted in larger PCR products than what expected, suggesting that there may be an intron within the amplicons, and PCR products of the other 33 primer pairs were smaller than expected, suggesting the occurrence of deletion within the genomic sequences or a lack of specificity or the possibility of assembly errors.

**Table 2:** Summary of cSSR searching results

Searching Item	Numbers
Total number of sequences examined	56,516
Total size of examined sequences (bp)	32,852,951
Total number of identified cSSRs	4,114
Number of cSSR containing sequences	3,594
Number of sequences containing more than 1 cSSR	423
Number of cSSRs present in compound formation	275
Di-nucleotide	1782
Tri-nucleotide	1747
Tetra-nucleotide	330
Penta-nucleotide	142
Hexa-nucleotide	113

In addition, the frequency, type and distribution of the potential 4,114 cSSRs were also analyzed in this study. The compilation of all cSSRs revealed that, on the average, one cSSR can be found every 7.99 kb in unigenes, and the frequency of cSSR was 7.78%. Among the 4,114 cSSRs, the di-and tri-nucleotide repeat motifs were the most abundant types (1,782, 43.32%; 1,747, 42.46%, respectively), followed by tetra- (330, 8.02%), penta- (142, 3.45%) and hexa-nucleotide (113, 2.75%) repeat motifs. Di- to hexa-nucleotide motifs were further analyzed for cSSR length (or number of repeat units, Table 3). cSSR length was mostly distributed from 12 to 20 bp, accounting for 83.76% of total cSSRs, followed by 21 - 30 bp length range (638 cSSRs, 15.51%). There were 30 cSSRs with length larger than 30 bp.

**Table 3:** Length distribution of cSSRs based on the number of repeat units

Number of repeat unit	Di-	Tri-	Tetra-	Penta-	Hexa-
4	0	0	219	115	100
5	0	1079	84	23	12
6	604	403	16	3	1
7	411	147	5	1	0
8	254	69	2	0	0
9	162	35	2	0	0
10	115	5	0	0	0
11	78	4	0	0	0
12	95	1	1	0	0
13	38	1	1	0	0
14	9	2	0	0	0
≥15	16	1	0	0	0

**Figure 9:**
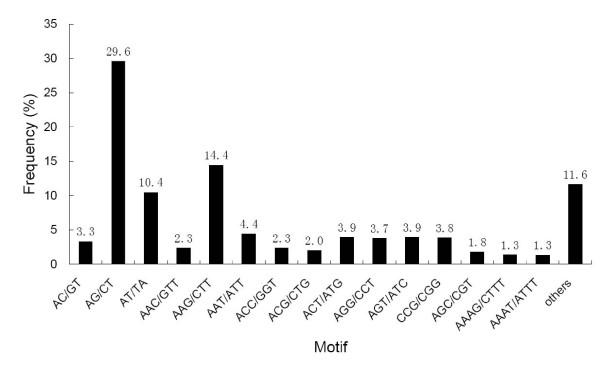
**Frequency distribution of cSSRs based on motif sequence types**. Within the searched cSSRs, a total of 160 motif sequence types were identified, of which, di-, tri-, tetra-, penta- and hexa-nucleotide repeat existed 4, 10, 30, 57 and 59 types, respectively. The AG/CT di-nucleotide repeat motif was the most abundant motif detected in our cSSRs.

Within the searched cSSRs, 160 motif sequence types were identified, of which, di-, tri-, tetra-, penta- and hexa-nucleotide repeat had 4, 10, 30, 57 and 59 types, respectively. The AG/CT di-nucleotide repeat was the most abundant motif detected in our cSSRs (1,216, 29.6%), followed by the motif AAG/CTT (593, 14.4%), AT/TA (429, 10.4%), AAT/ATT (180, 4.38%), ACT/ATG (159, 3.9%), AGT/ATC (159, 3.9%), CCG/CGG (157, 3.8%), AGG/CCT (154,3.7%) and AC/GT (135, 3.3%). The frequency of remaining 151 types of motifs accounted for 22.6% (Figure 9).

## Discussion

### Illumina paired end sequencing and assembly

Transcriptome sequencing is one of the most important tools for gene discovery. However, large-scale EST sequencing using the traditional Sanger method is time-consuming and expensive. During the past several years, the NGS technology has become a tremendous approach for high-throughput gene discovery on a genome-wide scale in non-model organisms. In addition to its great improvement of efficiency and speed, NGS platforms can eliminate the bacterial cloning step that can bias the composition of the cDNA library. Due to its long read length and appearance ahead of the other two platforms, Roche GS FLX has been the most widely used platform for *de novo *transcriptome sequencing in many organisms, such as chestnut [19], pine [10], olive [44], ginseng [20], *A. thaliana *[45,46], maize [47], *Artemisia annua *[48], fish [18], insects [49,50], and worms [17]. In contrast, Illumina transcriptome or genome sequencing was mainly limited to organisms with reference genomes available [14,51-53]. Over the last two years, with the further confirmation that the relatively short reads can be effectively assembled [15], especially with the great advantage of paired-end sequencing [54], the Illumina transcriptome or whole genome *de novo *sequencing and assembly have been successfully used for model [12,16,55-58]and non-model organisms [25-27,59]. Consistent with these publications, our results also indicated that relatively short reads from Illumina paired-end sequencing can be effectively assembled and used for novel gene discovery and SSR marker development in non-model organism. Here, approximately 59 million of 75-bp paired-end reads were generated from Illumina Genome Analyzer IIx. Such great numbers of reads and paired-end information resulted in a relatively high depth of coverage (average = 48.36 x). These sequences also produced longer unigenes (mean = 581 bp) than those assembled in previous studies for example, butterfly (197 bp) [60], *Eucalyptus grandis *(247 bp) [11], coral larval (440 bp) [17], lodegpole pine (500 bp) [10]) and whitefly (clusters: 372 bp; singletons: 265 bp) [27]).

In this study, we also provided evidence that a paired-end strategy had a strong impact on assembly as opposed to single-end approach. During *de novo *assembly, contigs were assembled from 75 bp reads data excluding mate-pair information, however, unigenes were assembled from the same read data combining with the mate-pair information. A comparison between contigs and unigenes revealed that both average and maximum lengths of unigenes were greatly larger than those of contigs, though the number of contigs was more than that of unigenes (Table 1). This illustrated the critical importance of reads pairs for obtaining high-quality assemblies. The high quality assembled unigenes were validated by a high proportion of unigenes matching to known proteins using BLASTx and by the efficient PCR amplification of cSSR markers developed in our unigenes.

Nonetheless, only 40% of reads were assembled into unigenes, which is less than that reported for 454 transciptome assemblies (e.g., 88% [11], 90% [17], 48% [10]). Large numbers of un-assembled reads could result from several causes, including the relatively short reads generated by Illumina Genome analyzer, the assembly options (e.g., the K-mer size), genes expressed at low levels, repeat regions, and the difficulties with *de novo *transcriptome assembly caused by the alternative splicing. These high-quality unassembled reads are still a very important sequence resource for sweetpotato. To combine the use of longer reads for example from the FLX-454 sequencing platform would possibly further improve the *de novo *assembly.

When we realigned all the usable sequencing reads to the unigenes, a 48 × average coverage depth was obtained. However, of the 56,516 unigenes, 406 (0.7%) had a coverage depth less than 1. This is in part due to the drawback to the de Bruijn graph approach [61], which is the algorithm used by SOAPdenovo. In de Bruijn approach, the reads are decomposed into k-mers, which maybe cause the loss of information. In a few cases, only partial K-mers from the reads can be used for assembly, leading to assembled sequences that are not supported by the underlying reads. This also implied that the paralogs that share a high level of sequence similarity may have been assembled into one contig because they can not be distinguished due to the short read length and the lack of reference genome.

### Functional annotation of unigenes

Estimating the number of genes and the level of transcript coverage is an important issue for transcriptome sequencing projects, but is difficult in this study due to the lack of a reference genome. Using BLAST, we indirectly evaluated the transcriptome coverage breadth by determining the number of unique genes in our collection. A great number of unigenes could match unique known proteins in public databases, which implied that our Illumina paired-end sequencing project yielded a substantial fraction of unique genes from sweetpotato. Like [10,19], if we assumed that the number of genes in sweetpotato was commensurate with that in *Arabidopsis *(25,000 genes, [62]), our annotated unigenes (20,755 genes with unique protein accession numbers) would likely represent more than 80% of genes in sweetpotato. A large number of unigenes were assigned to a wide range of gene ontology categories and COG classifications (Figure 6, 7), also indicating that our paired-end sequencing data represented a wide diversity of transcripts. Based on the KEGG pathway, the well represented pathways were carbohydrate metabolism, biosynthesis of secondary metabolite, amino acid metabolism, lipid metabolism and energy metabolism (Figure 8A). In the secondary metabolism, 2,493 unigenes were classified into 19 different subcategories (Figure 8B). These results indicated the active metabolic processes in sweetpotato root development. Notably, we also found all of the genes involved in the biosynthesis of brassinosteroid (pathway not shown). We estimated that the expression of brassinosteroid biosynthetic genes was lower than that of the genes involved in the biosynthesis of starch and sugar. Therefore, these results also strongly suggested that most of the genes involved in the different metabolic processes came into being through high-throughput Illumina transcriptome sequencing. Furthermore, the unigenes without BLAST hits likely corresponded to 3' or 5' untranslated regions, non-coding RNAs, or short sequences not containing a known protein domain, most of which might represent potential sweetpotato-specific genes. Taken together, such large number of sequences and deep depth of coverage can provide sufficient transcriptomic sequence information for discovering novel genes, and also confirm that high throughput Illumina paired-end sequencing is an efficient, inexpensive and reliable tool for transcriptome characterization and gene discovery in non-model species. Generally speaking, cDNA normalization is often used when gene discovery is the primary purpose of sequencing. According to previous publication, there is no real advantage to normalization when thousands of sequences were generated [18]. In this study, such deep depth and wide breadth of coverage provided by the powerful Illumina paired-end sequencing platform suggested that it was feasible to obviate the need for normalization.

### cSSR marker identification and characterization

In this study, a total of 100 pairs of high quality PCR primers were designed and used for further assessment of the assembly quality. Of these, 92 (92%) could successfully yield amplicons. Among the 92 working primer pairs, 47 amplified PCR products at the expected sizes, and 45 primer pairs resulted in larger or smaller PCR products than what expected, suggesting that there may be an intron or deletion within the amplicons or a lack of specificity, it also can not ruled out the possibility of assembly errors due to the short read length. This result was similar to previous studies in which success rates of 60-90% amplification have been reported [63-67], and also provided evidence for the quality validation of our assembled unigenes and the possibility of the utility of the cSSRs produced in the present study.

As is commonly known, polymorphic SSR markers are important for research involving genetic diversity, relatedness, evolution, linkage mapping, comparative genomics, and gene-based association studies. Next generation transcriptome sequencing produces plenty of sequences for molecular marker development. Currently there exist only several hundreds genetic markers in sweetpotato. The 4,114 cSSRs identified from our data will provide a wealth of markers for further genetic study. Based on these identified cSSR-containing sequences, we will design more PCR primers and assess their polymorphism among cultivated and wild *Ipomoea *species and provide a more valuable resource of genetic markers for future research in sweetpotato.

## Conclusion

In this study, in addition to the characterization of the root transcriptome of sweetpotato, we achieved some valuable resources for new gene discovery and cSSR marker development for further study. Many genes generated in the present study will certainly accelerate the understanding of the processes regulating sweetpotato root formation and development. To the best of our knowledge, this is the first attempt using Illumina paired-end sequencing technology for sweetpotato root transcriptome *de novo *sequencing and assembly without reference genome. Additionally, in these generated sequences, 4,114 cSSRs were identified and characterized as potential molecular markers. The enormous size and complexity of sweetpotato genome make it essential to develop thousands of molecular markers for the fine-scale mapping of interest traits. Thousands of cSSR markers produced in this study will enable genetic linkage mapping construction and gene-based association studies. The results demonstrated that Illumina paired end sequencing can be used as a fast and cost-effective approach to the gene discovery and molecular marker development for non-model organism, especially those with large genome.

## Methods

### Plant material and RNA extraction

Sweetpotato cultivar "Guangshu 87" was grown in the experimental station of the Crops Research Institute, Guangdong Academy of Agricultural Sciences, Guangzhou, China. Samples were collected from fibrous roots (diameter <0.5 cm), pencil roots (diameter 0.5-1.2 cm) and tuberous roots at three developmental stages of growth: initial tuberous root (diameter 0.5-1.0 cm); swelling tuberous root (diameter 3.0-3.5 cm) and mature tuberous root (diameter >5.0 cm). The sampled tissues were immediately frozen in liquid nitrogen and stored at -80° until use.

For Illumina sequencing, the total RNA of each sample was isolated using a CTAB-based protocol and further purified with the RNeasy Plant Mini Kit (Qiagen, Valencia, CA). RNA quality was verified using a 2100 Bioanalyzer RNA Nanochip (Agilent, Santa Clara, CA) and all five samples had RNA Integrity Number (RIN) value more than 8.5. Then RNA was quantified using NanoDrop ND-1000 Spectrophotometer (NanoDrop, Wilmington, DE). A total of 20 μg of RNA was equally pooled from the five tissues for cDNA library preparation.

### cDNA library construction and sequencing

Illumina sequencing using the GAII platform was performed at Beijing Genomics Institute (BGI)-Shenzhen, Shenzhen, China http://www.genomics.cn/index.php according to the manufacturer's instructions (Illumina, San Diego, CA). Briefly, poly (A) RNA was isolated from 20 μg of total RNA using Sera-mag Magnetic Oligo (dT) Beads (Illumina). To avoid priming bias when synthesizing cDNA, the purified mRNA was first fragmented into small pieces (100-400 bp) using divalent cations at 94°C for exactly 5 minutes. Then the double-stranded cDNA was synthesized using the SuperScript Double-Stranded cDNA Synthesis kit (Invitrogen, Camarillo, CA) with random hexamer (N6) primers (Illumina). The synthesized cDNA was subjected to end-repair and phosphorylation using T4 DNA polymerase, Klenow DNA polymerase and T4 PNK. These repaired cDNA fragments were 3' adenylated using Klenow Exo- (3' to 5' exo minus, Illumina). Illumina Paired-end adapters were ligated to the ends of these 3'-adenylated cDNA fragments. To select a size range of templates for downstream enrichment, the products of ligation reaction were purified on a 2% TAE-agarose gel (Certified Low-Range Ultra Agarose, Biorad). A range of cDNA fragments (200 ± 25 bp) was excised from the gel. Fifteen rounds of PCR amplification were performed to enrich the purified cDNA template using PCR Primer PE 1.0 and PCR Primer PE 2.0 (Illumina)] with Phusion DNA Polymerase. The cDNA library was constructed with a fragment length range of 200 bp (±25 bp). Finally, after validating on an Agilent Technologies 2100 Bioanalyzer using the Agilent DNA 1000 chip kit, the cDNA library was sequenced on a PE flow cell using Illumina Genome Analyzer IIx, and the workflow was as follows: template hybridization, isothermal amplification, linearization, blocking, sequencing primer hybridization, and sequencing on the sequencer for Read 1. After completion of the first read, the templates can be regenerated *in situ *to enable a second 75 bp read from the opposite end of the fragments, i.e., the newly sequenced strands are stripped off and the complementary strands are bridge amplified to form clusters. Once the original templates are cleaved and removed, the reverse strands undergo sequencing-by-synthesis, producing 59,233,468 sequencing reads with 75-mer length. The sequencing data are deposited in NCBI Sequence Read Archive (SRA, http://www.ncbi.nlm.nih.gov/Traces/sra) [68] with accession number SRA022988.

### Data filtering and *de novo *assembly

The quality requirement for *de novo *transcriptome sequencing is far higher than that for re-sequencing, because sequencing errors can create difficulties for the short-read assembly algorithm. We therefore carried out a stringent filtering process. Firstly, we removed reads that do not pass the built-in Illumina's software Failed-Chastity filter according to the relation "failed-chastity < = 1", using a chastity threshold of 0.6, on the first 25 cycles. Secondly, we discarded all reads with adaptor contamination. Thirdly, we ruled out low-quality reads with ambiguous sequences "N". Finally, the reads with more than 10% Q < 20 bases were also removed.

*De novo *assembly was carried out using SOAPdenovo http://soap.genomics.org.cn/soapdenovo.html with the default settings except K-mer value [16]. After assessing different K-mer sizes, 29-mer yielded the best assembly for the desired application, and was chosen to construct the *de Bruijn *graph. Although this higher value reduced the number of assembled contigs, it increased the reliability and longer contigs. The contigs without N were obtained by conjoining the *K*-mers in an unambiguous path. Then the reads were mapped back to contigs for constructing scaffolds with the paired end information. SOAPdenovo connected the contigs using N to represent unknown sequences between each two contigs, and thus scaffolds were made. Paired-end reads were used again for gap filling of scaffolds to get sequences with least Ns and could not being extended on either end. Such sequences were defined as Unigenes. To evaluate the depth of coverage, all usable reads were realigned to the unigenes using SOAPaligner (Release 2.20, 08-13-2009) [28] with the default settings except the following changes, -m 0 -x 1000 -s 40 -l 35 -v 2. Detailed options could be found at this website http://soap.genomics.org.cn/soapaligner.html.

Finally, BLASTx alignment (*E *value <10^-5^) between unigenes and protein databases like NCBI non-redundant protein (Nr) database http://www.ncbi.nlm.nih.gov, Swiss-Prot protein database http://www.expasy.ch/sprot, the Kyoto Encyclopedia of Genes and Genomes (KEGG) pathway database http://www.genome.jp/kegg, and the Cluster of Orthologous Groups database http://www.ncbi.nlm.nih.gov/COG was performed, and the best aligning results were used to decide the sequence direction of unigenes. If the results of different databases conflicted with each other, a priority order of Nr, Swiss-Prot, KEGG and COG should be followed when deciding the sequence direction of unigenes. When a unigene happened to be unaligned to none of the above databases, a software named ESTScan [69] was used to predict its coding regions as well as to decide its sequence direction.

### Gene annotation and analysis

To assess the quality of the *de novo *assembly through comparative genome analysis, a similarity search against *A. thaliana *gene index was conducted using BLASTN algorithm with *E *value less than 10^-5^. The *A. thaliana *gene index (version 15) was downloaded from TIGR gene indices (currently curated at Harvard University, http://compbio.dfci.harvard.edu/tgi/). The BLAST result was parsed by a Perl script written based on the bioperl module SearchIO.pm.

For further annotation of unigenes using various bioinformatics approaches, the unigenes were firstly searched against the Nr database and the Swiss-Prot protein database using local BLASTx with *E *value cutoff of 10^-5^. To estimate the number of annotated unigenes that matched to unique genes in the two databases, these files were then filtered for the duplicate in protein accessions. With Nr annotation, Blast2GO program [42] was used to get GO annotation according to molecular function, biological process and cellular component ontologies http://www.geneontology.org. The unigene sequences were also aligned to the COG database to predict and classify possible functions. Pathway assignments were carried out according to the Kyoto Encyclopedia of Genes and Genomes pathway database [70] also using BLASTx with *E *value threshold of 10^-5^.

### Development of cDNA-derived SSR markers

A Perl script known as MIcroSAtellite (MISA, http://pgrc.ipk-gatersleben.de/misa/) was used to identify microsatellites in the unigenes. In this study, cDNA-based SSRs were considered to contain motifs with two to six nucleotides in size and a minimum of 4 contiguous repeat units. Frequency of cSSR refers to kilobase pairs of cDNA sequences containing one SSR. Primer premier 6.0 (PREMIER Biosoft International, Palo Alto, CA) was used to design PCR primers in the flanking regions of SSRs. Primers were designed based on the following criteria: (1) primer length with a minimum of 18 bp long; (2) melting temperature between 46°C and 55°C with a maximum discrepancy within 4°C among primers; (3) PCR product size ranging from 100 to 350 bp. In total, we designed 100 pairs of primers (Additional file 2, Table S2) and tested these primer sets for successful PCR amplification in initial screening test.

## Authors' contributions

ZYW conceived, organized and planned the research, contributed to RNA extraction and data analysis, and drafted the manuscript. BPF conceived this study and participated in design, coordination and manuscript preparation. JYC provided the plant material for transcriptome sequencing and participated in tissue collections. XJZ provided the plant materials for SSR analysis. ZXL helped to design PCR primers and SSR results analysis. LFH participated in RNA and DNA extraction. XLC participated in RNA and DNA extraction and SSR experiment. YJL participated in manuscript preparation and revision. All authors read and approved the final manuscript.

## Supplementary Material

Additional file 1 **Table S1**. The most abundant unigenes in Illumina sequencing data. Click here for file

Additional file 2 **Table S2**. Primer sequences for cSSR Markers. Click here for file
