# Supplementary material for: De novo assembly and characterization of root transcriptome using Illumina paired-end sequencing and development of cSSR markers in sweetpotato (Ipomoea batatas)
Source: BMC Genomics. 2010 Dec 24;11:726. doi: 10.1186/1471-2164-11-726 (PMC3016421; doi:10.1186/1471-2164-11-726)
Supplement: Additional file 1 — Table S1. The most abundant unigenes in Illumina sequencing data. [file 1471-2164-11-726-S1.DOC]

| **Unigene ID** | **No. of reads** | **ID** | **E-Evalue** | **Putative annotation** | **Source** |
| --- | --- | --- | --- | --- | --- |
| Unigene15030 | 49469 | No hit |  |  |  |
| Unigene4520 | 30626 | No hit |  |  |  |
| Unigene25072 | 27731 | gi|240254647| | 0 | transferase, transferring glycosyl groups | *Arabidopsis thaliana* |
| Unigene17641 | 27303 | gi|586004|sp| | 4E-60 | RecName: Full=Superoxide dismutase [Cu-Zn] | *Ipomoea batatas* |
| Unigene11559 | 22371 | P10537 | 1E-102 | AMYB_IPOBA Beta-amylase | *Ipomoea batatas* |
| Unigene2261 | 14541 | gi|167859793| | 4E-41 | senescence-associated protein | *Picea abies* |
| Unigene1085 | 13927 | gi|57013900| | 2E-08 | hypothetical protein NitaMp027 | *Nicotiana tabacum* |
| Unigene6116 | 13721 | P33444 | 1E-103 | RS3A_CATRO 40S ribosomal protein S3a | *Catharanthus roseus* |
| Unigene20938 | 13116 | gi|62701389| | 4E-11 | metallothionein-like type 2 protein | *Ipomoea batatas* |
| Unigene17452 | 11927 | gi|89276319| | 2E-48 | ribosomal protein | *Gymnadenia conopsea* |
| Unigene25225 | 11153 | gi|86438862| | 2E-18 | putrescine N-methyltransferase | *Calystegia sepium* |
| Unigene24422 | 10807 | gi|126363759| | 2E-32 | ADP-glucose pyrophosphorylase beta subunit  IbAGPb1A | *Ipomoea batatas* |
| Unigene18197 | 10123 | gi|242070913| | 7E-22 | hypothetical protein SORBIDRAFT_05g016477 | *Sorghum bicolor* |
| Unigene2202 | 10036 | gi|13491754| | 6E-17 | metallothionein-like protein | *Ipomoea batatas* |
| Unigene56438 | 9541 | No hit |  |  |  |
| Unigene55912 | 9077 | Q38912 | 1E-105 | RAC3_ARATH Rac-like GTP-binding protein | *Arabidopsis thaliana* |
| Unigene1209 | 9076 | gi|87240819| | 6E-31 | Like-Sm ribonucleoprotein-related, core | *Medicago truncatula* |
| Unigene28362 | 9054 | gi|145408590| | 6E-18 | ORF124 | *Pinus koraiensis* |
| Unigene56497 | 9002 | No hit |  |  |  |
| Unigene14059 | 8872 | gi|7959366| | 3E-12 | ribosomal protein L29 | *Panax ginseng* |
| Unigene52889 | 8862 | gi|290490154| | 1E-100 | ATP synthase CF1 beta subunit protein | *Meliosma aff. Cunei- folia Moore 333* |
| Unigene56391 | 8762 | No hit |  |  |  |
| Unigene5831 | 8291 | P27598 | 3E-61 | Alpha-1,4 glucan phosphorylase L isozyme, chloroplastic/amyloplastic | *Ipomoea batatas* |
| Unigene47266 | 8139 | Q3TLS3 | 2E-17 | CO058_MOUSE UPF0580 protein C15orf58 homolog | *Mus musculus* |
| Unigene28158 | 8131 | gi|284437921| | 3E-44 | GDP-D-mannose 3',5'-epimerase | *Actinidia deliciosa* |
| Unigene7089 | 8082 | gi|158938836| | 2E-25 | granule bound starch synthase I | *Ipomoea batatas* |
